# Noxious effects of riot control agents on the ocular surface: Pathogenic mechanisms and management

**DOI:** 10.3389/ftox.2023.1118731

**Published:** 2023-01-17

**Authors:** Manuel E. Quiroga-Garza, Raul E. Ruiz-Lozano, Nadim S. Azar, Hazem M. Mousa, Seitaro Komai, Jose L. Sevilla-Llorca, Victor L. Perez

**Affiliations:** ^1^ Department of Ophthalmology, Duke University Medical Center, Durham, NC, United States; ^2^ Foster Eye Center for Ocular Immunology, Duke Eye Center, Durham, NC, United States; ^3^ Tecnologico de Monterrey, School of Medicine and Health Sciences, Institute of Ophthalmology and Visual Sciences, Monterrey, Mexico

**Keywords:** riot control agents, tear gas, toxicity, oleoresin capsicum, chlorobenzylidene malononitrile, neurogenic inflammation, ocular surface

## Abstract

Riot Control Agents (RCAs) are chemical compounds used by law enforcement agencies to quell violent demonstrations as an alternative to lethal force and as part of police/military training. They are also known as tear gases because of the hallmark ocular irritation and lacrimation they cause. The most common RCAs include oleoresin capsicum (contained in Mace and pepper spray), chlorobenzylidene malononitrile, dibenzoxazepine, and chloroacetophenone (previously the main content of Mace); some of which have been in use for decades. Their immediate incapacitating effects are mediated through polymodal afferent fibers innervating the corneal surface, inducing the release of peptides that cause neurogenic inflammation. Although previously thought to have only transient effects on exposed patients more severe complications such as corneal stromal opacities, corneal neovascularization, neurotrophic keratopathy, conjunctival necrosis, and pseudopterygium can occur. Concerningly, the lack of research and specific therapies restrict the current management to decontamination and symptom-tailored support. This manuscript will provide an overview of the toxic mechanisms of RCAs, their clinical manifestations, and current therapy after exposure to tear gases.

## 1 Introduction

Riot control agents (RCAs), also known as chemical crowd control agents, are chemical agents that cause temporary disability, usually a little longer than the exposure period (Menezes et al., 2016). They represent a non-lethal and non-confrontational alternative for authorities to pacify large crowds causing a civilian disturbance or curtail advancing enemy military forces (Toprak et al., 2015). To manage violent crowds, the ideal RCA has a rapid onset of action, a brief duration of effects, and a good safety profile to avoid permanent damage (Kim et al., 2016). In contrast, to hinder the advancement of a military force, the chemical should ideally remain in the environment for weeks to months (Menezes et al., 2016). Due to their ease of use and immediate onset of action, aerosolized chemicals, the so-called tear gases, are the most frequently used RCAs, including chloroacetophenone (CN), oleoresin capsicum (OC), dibenzoxazepine (CR), and chlorobenzylidene malononitrile (CS) (Brown et al., 2000; Zollman et al., 2000; Yeung and Tang, 2015). These chemicals are the main constituents of pepper sprays, and CN was the active compound in the original formula of the product marketed as “Mace” for self-defense use or as an animal deterrent (bear mace). However, OC and CS alone or in combination have replaced CN in modern formulations due to less toxic effect profiles (Smith and Greaves, 2002; Kearney et al., 2014). To this day, exposure to these agents is part of the training regime used in some law enforcement academies.

Tear gases rapidly disable the victim by inflicting damage to the eye’s ocular surface, the outermost part (Krishnatreyya et al., 2018b). The extent of the damage varies depending on the form of delivery. In the acute setting, aerosolized agents may cause lacrimation, erythema, conjunctival edema, blurred vision, and eye pain (Dimitroglou et al., 2015). In contrast, explosive weapons may cause thermal, chemical, and physical damage imposed by the blast (MacLeod, 1969; Tidwell and Wills, 2019). If left untreated, tear gases may lead to permanent vision loss due to conjunctival scarring and loss of corneal sensation leading to neovascularization, stromal thinning, ulceration, infection, and perforation (Levine and Stahl, 1968). Although rare, blindness could also result from secondary glaucoma, cataract formation, vitreous hemorrhage, and traumatic optic neuropathy (Kim et al., 2016). Thus, acute management and careful follow-up are necessary after eye exposure to RCAs to avoid sight-threatening complications.

The ramifications that arise from the use of these substances are not only limited to the eyes. The respiratory system is the other main target of RCAs, but dermatological, gastrointestinal, and even neurologic symptomatology can be observed (Hu et al., 1989; Dimitroglou et al., 2015). Depending on the concentration used and the length of exposure, manifestations range from copious rhinorrhea, sneezing, salivation, and skin erythema to more severe complications like laryngeal edema, pulmonary edema, chemical burns, and panic attacks (Vaca et al., 1996; Varma and Holt, 2001). Some of these exposures have proven to be lethal (Haar et al., 2017). Considering that the eyes are one of the main targets of RCAs, it is crucial for clinicians to possess knowledge of how these patients could present. This review aims to provide an up-to-date overview of the clinical presentation, pathogenic mechanisms, and treatment of ocular surface toxicity induced by RCAs.

## 2 Historical background and epidemiological data

The use of poisonous gases was reported as early as 430, 431 BC when Spartans released irritating gases of coal, burning wax, and pitch to the environment during the Peloponnesian War against the Athenians (Sanford, 1976). During World War I (1914–1918 AC), the German army was the first to use chemical agents that caused temporal disability by producing excessive blepharospasm and lacrimation, including chloropicrin, benzyl bromide, and acrolein, among others. By the early 1920s, civilians could purchase pocket-size tear gas devices containing CN to carry for self-defense purposes (Sanford, 1976; Frey et al., 2022). In 1925, in Geneva, the Protocol for the Prohibition of the use in War of Asphyxiating, Poisonous or Other Gases, and of Bacteriological Methods of Warfare was signed under the auspices of the (World Health Organization, 1970). Despite the latter, the United States signed an executive order in 1975 that allowed using RCAs in certain situations, including control of war prisoners and convoy protection outside combat zone; thus, they do not consider RCAs as warfare agents (Frey et al., 2022).

During 1998–2002, the Texas poison centers reported 1,531 human pepper spray (OC) exposures (Forrester and Stanley, 2003). Of those, 84% were unintentional, 68% occurred in the house, 64% involved children and teenagers, and 56% occurred in men (Forrester and Stanley, 2003). In 2017, the National Poison Data System (NPDS) reported 4,007 total exposures to tear gases, including OC (83%), CN (12%), CS (0.2%), and others (4%) (Gummin et al., 2018). Although 25% of the cases were treated in a health care facility, only 0.12% of victims suffered major adverse outcomes (Gummin et al., 2018).

## 3 Delivery systems of riot control agents (RCAs)

RCAs are usually referred to as “tear gases.” However, rather than a gas, they are compounded as an aerosol of solid particles (Rothenberg et al., 2016). They may be projected from solutions or as airborne dispersions. While the former includes personal defense sprays and gas cartridges, canisters, and grenades employed by law enforcement (Ilgaz et al., 2019), the latter contains dispersions generated as smokes, aerosol mists, or powder clouds (Ballantyne, 2006). Hand-held devices contain liquid formulations released through narrow or wide-angle pressurized sprays to incapacitate one person (Schep et al., 2015). On the other hand, canisters and grenades are a pyrotechnic mixture blended with a powder form that is aerosolized for dispersion as smokes (Olajos and Stopford, 2004; Rothenberg et al., 2016). These tear gas pyrotechnic devices can engage targets as far as 300 m^2^, ideal for crowd control in riots (Rothenberg et al., 2016). Aircraft, vehicle, and drone-guided technologies are also used as delivery systems. Additionally, non-lethal projectile weapons have a high risk of inducing severe traumatic injuries when fired at a person (Ifantides et al., 2020).

## 4 Anatomical features

The cornea is the most densely innervated tissue in the body. The ophthalmic branch (V_1_) of the trigeminal nerve oversees the nociceptive functions of the eye, including the blinking reflex, tear production, and wound healing (Ruiz-Lozano et al., 2021). The nasociliary nerve, a V_1_ branch, enters the orbit to cover the ocular surface. Nasociliary nerve branches decussate, pierce the sclera, and travel anteriorly to innervate the corneoscleral limbus and the corneal stroma (Marfurt et al., 2010). Subsequently, they form the subepithelial plexus and cross the Bowman’s membrane to form the subbasal nerve plexus, which innervates the corneal epithelium (Marfurt et al., 2010; Ruiz-Lozano et al., 2021). There are three types of sensory corneal nerves, all of which evoke pain. They are classified into polymodal nociceptor neurons, pure mechanoreceptors, and cold thermoreceptors based on the activating noxious stimuli (Belmonte et al., 2015; Belmonte et al., 2017). The mechanism of action of tear gases occurs due to the activation of transient receptor potential (TRP) ion channels, a group of sensitizing chemosensory receptors located in peripheral nerve endings (Rothenberg et al., 2016; Frey et al., 2022). The TRP vanilloid (TRPV1), an agonist of OC, also known as capsaicin, and ankyrin (TRPA1) agonist of CS, CN, and CR, are two subfamilies of TRP ion channels (Schep et al., 2015). They are both expressed in the peripheral pain-sensing nociceptive nerves of the skin, the mucous membranes of the lung and upper and lower airways, and the ocular surface (cornea and conjunctiva) (Rothenberg et al., 2016).

## 5 Ocular surface toxicity of specific chemicals used as riot control agents (RCAs)

### 5.1 Oleoresin capsicum (OC)

#### 5.1.1 Chemical properties

OC is a mixture of fat-soluble phenols (capsaicinoids) obtained from the pepper plants *Capsicum frutescens* and *Capsicum annuum* (Ballantyne, 2006). Capsaicin (C_18_H_27_NO_3_), the main component of OC, has a melting and boiling point of 64°C and 210–220°C, respectively, and a molecular weight of 305.41. The threshold for ocular irritation is 0.002 mg/m^3^ (Schep et al., 2015; Kim et al., 2016). The concentration of OC in pepper sprays varies between manufacturers (1.2%–12.6%) (Ballantyne, 2006).

#### 5.1.2 Mechanism of toxicity

**FIGURE 1:**
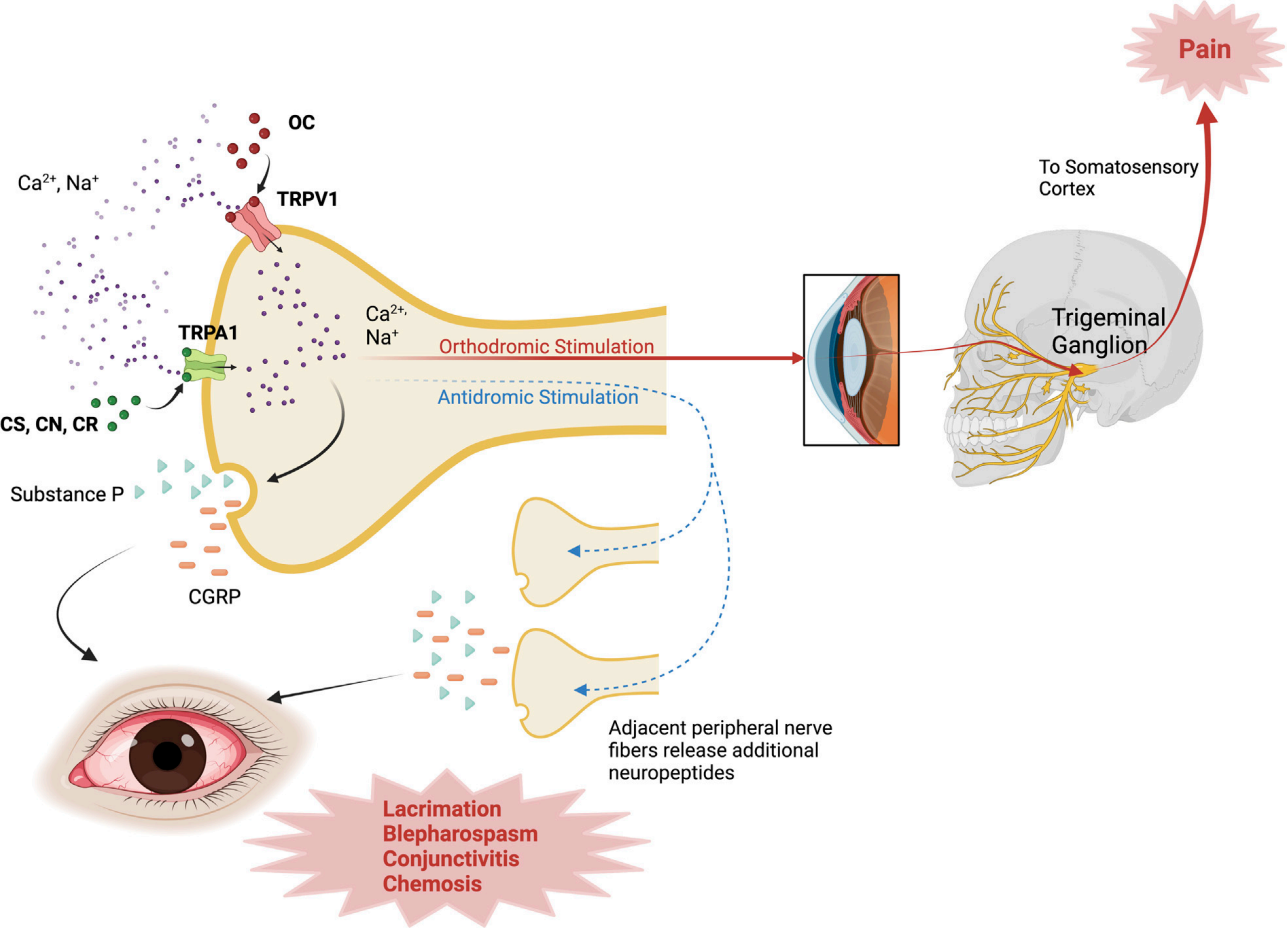
Mechanism of action of Riot Control Agents. OC activates TRPV1 channels; while CS, CN, CR activate TRPA1 channels in corneal sensory neurons, inducing Ca^2+^ influx. With orthodromic stimulation, the impulse travels through the trigeminal pathway to the somatosensory cortex and elicits the pain sensation. In antidromic stimulation, TRP channel activation triggers an impulse that travels to adjacent nerve fibers, inducing the release of additional neuropeptides (i.e., SP, CGRP) to propagate inflammation. CGRP, Calcitonin-Gene Related Peptide; CN, chloroacetophenone; CR, Dibenzoxazepine; CS, chlorobenzylidene malononitrile; TRPA1, Transient Receptor Potential Ankyrin 1; TRPV1, Transient Receptor Potential Vanilloid 1. Created with Biorender.com.

Capsaicin has agonistic activity at TRPVI, a non-selective channel permeable to calcium and sodium in corneal sensory neurons (Belmonte et al., 2017; Alamri et al., 2018). Upon painful stimuli with OC, the TRPV1 channel opens, allowing calcium entry with subsequent channel inactivation and resulting analgesia (Bates et al., 2010). Besides pain, OC stimuli also trigger an inflammatory response, the so-called neurogenic inflammation. This process also involves membrane depolarization through non-selective channel opening, thus increasing intracellular calcium and sodium, allowing the release of neuropeptides by polymodal nociceptive neurons such as substance P and calcitonin-gene-related peptide (Kumar et al., 2018). The neurogenic inflammation model involves the axon-reflex hypothesis, where depolarization of the afferent fiber triggers an action potential traveling in one direction to the CNS to elicit the pain sensation (orthodromic stimulation); additionally, at axonal branch-points, an opposite-direction nervous impulse induces the release of neuropeptides from nearby afferent nerve endings to potentiate inflammation (antidromic stimulation) (Yeung and Tang, 2015; Sorkin et al., 2018). A schematic representation of these mechanisms is found in Figure 1.

In a rabbit model, Gallar et al. demonstrated delayed corneal wound healing after topical and retrobulbar capsaicin application, suggesting damage to trigeminal nerve fiber endings and neuropeptide depletion (Gallar et al., 1990). In a murine model, Lambiase et al. observed a significant decrease in corneal innervation, peripheral sensitivity, corneal healing rate, and tear secretion after subcutaneous injection of capsaicin (Lambiase et al., 2012). After epithelial debridement, the authors report a significant decrease in nerve growth factor (NGF), a crucial factor that oversees the proliferation and survival of sensory neurons (Lambiase et al., 2012).

#### 5.1.3 Ocular manifestations

Ocular irritation can occur with small capsaicin particles (2 µm), whereas severe and prolonged irritation occurs with more extensive (50 µm) particles. Although the lipid-soluble properties of capsaicin confer the ability to penetrate the corneal epithelium easily, its poor water solubility avoids damage to deeper corneal layers (Krishnatreyya et al., 2018a). In 47 cadets, Zollman et al. reported conjunctival injection, variable pain, and blepharospasm in all cases, punctate epithelial erosions (PEE, 21%), and a significant reduction in corneal sensitivity, measured with the Cochet-Bonnet esthesiometer 10 min after exposure to pepper spray (5.7 ± 0.4 cm vs. 0.6 ± 1.0 cm) during a training exercise (Zollman et al., 2000). After 1-week, the PEE was healed, and the corneal sensation was restored (Zollman et al., 2000). Vesaluoma et al. also found decreased corneal sensitivity and transient *in vivo* confocal microscopy (IVCM) changes, including corneal epithelial swelling, in ten police officers exposed to OC in a controlled setting (Vesaluoma et al., 2000).

Although results from the previous studies suggest that OC is harmless to the ocular surface, in all of them, exposure occurred in a controlled setting. Sustained corneal abrasions occurred in 7% of subjects in a jail’s emergency department exposed to pepper spray at a 10% concentration (Brown et al., 2000). Holopainen et al. reported deep conjunctival and corneal damage that partially resolved after weeks to months in four cases exposed to pepper sprays, three containing OC. One case was only exposed to the solvent, suggesting the latter also causes ocular surface toxicity caused by OC sprays (Holopainen et al., 2003). IVCM findings revealed keratocyte activation in the deep corneal stroma of one case (Holopainen et al., 2003). Another study reported a significant reduction in tear production, measured with the Schirmer test, and dry eye symptoms 2 weeks after exposure to pepper spray in patients during a public protest in Turkey (Rasier et al., 2015). A 75-year-old man developed severe conjunctival chemosis with necrosis, symblepharon formation, and a subtotal corneal epithelial defect after exposure to topical capsaicin (Das et al., 2005).

### 5.2 Chloroacetophenone (CN)

#### 5.2.1 Chemical properties

CN, also known as phenylacyl chloride and *α*-CN, was developed after World War I and has been used for riot control and self-defense. However, severe adverse effects are reported with its use, including death due to pulmonary asphyxia (Chapman and White, 1978; Ballantyne, 2006). Thus, countries like the United Kingdom no longer use CN for peacekeeping operations. It is still used in the United States (Ballantyne, 2006). CN (C_8_H_7_ClO) has a melting and boiling point of 58°C–59°C and 244°C–245°C, respectively, and a molecular weight of 154.59. It is soluble in ether, ethanol, and benzene and insoluble in water (Schep et al., 2015). The threshold for ocular irritation is 1.0 mg/m^3^. CN is sold as MACE^®^, a 1% CN solution in a solvent of 5% 1,1,1-trichloroethane, 4% kerosene, and Freon 113 (Blain, 2003). It is a micro-pulverized powder that can cause thermal and mechanical due to the force of the blast and chemical damage to the eye (Levine and Stahl, 1968). The half-maximal activation of TRPA1 induced by CN is EC_50_ CN = 91 ± 12 nM (Bessac et al., 2009).

#### 5.2.2 Mechanism of toxicity

CN and CS (See Section 5.3) are SN_2_-alkylating agents that react with nucleophilic sites, the former tenfold more potent. The studies performed by Ballantyne and Swanston in 1978 determined that the toxicity induced by CN is caused by the inactivation thiol and sulphydryl-containing enzymes, including pyruvic decarboxylase and glutamic dehydrogenase (Blain, 2003; Ballantyne, 2006). Additionally, CS can also reversibly inhibit lactate dehydrogenase, while CN cannot (Sanford, 1976). Therefore, some of the toxic effects caused by these RCAs are caused of the disruption of intracellular metabolic pathways including glycolysis and the tricarboxylic acid cycle (Mackworth, 1948; Castro, 1968). Studies on animal models showed that CN was the more toxic compound in comparison to CS, demonstrated by the higher rate of lethal tissue damage in small mammals (Ballantyne and Swanston, 1978). This reaction causes the degradation of enzymes related to sensory nerve activity (Levine and Stahl, 1968). The TRPA1 receptor, another cationic channel permeable to calcium, sodium, and potassium, is also present in polymodal nociceptor neurons and, thus, can be activated by chemical stimuli (Kaneko and Szallasi, 2014; Ruiz-Lozano et al., 2021). TRPA1 receptors contain nucleophilic groups (i.e., cysteine thiols) that form covalent interactions with CN, CS, and CR, potent agonists of these receptors (Bautista et al., 2006; Brône et al., 2008). The transcription of the TRPA1 gene has been found in the trigeminal neurons, dorsal root ganglion neurons, and corneal nerves of mice, rats, and humans (Brône et al., 2008; Canner et al., 2014). Bessac et al. reported absent or minimal response to pain in mice with genetic ablation or pharmacological blockade of TRPA1, confirming the role of TRPA1 in pain detection (Bessac et al., 2009). Corneal expression of TRPA1 is also related to transforming growth factor (TGF)-β1 fibrotic responses, as TRPA−/−mice corneas remained more transparent after alkali-burned injury (Okada et al., 2015). Furthermore, TRPA1 activation also leads to increased corneal levels of substance P, which facilitates a lower neuronal threshold of activation that sensitizes the cornea to further stimuli, including non-noxious ones (Zhang et al., 2007).

#### 5.2.3 Ocular manifestations

In a rabbit model, Ballantyne et al. reported lacrimation, purulent discharge, blepharitis, conjunctival chemosis, increased intraocular pressure (IOP), hyperemia, iritis, keratitis, and corneal neovascularization after conjunctival sac instillation of 0.1 mL of CN dissolved in polyethylene glycol 300 (PEG300) at concentrations ranging from 1% to 10% (Ballantyne et al., 1975). The severity and duration of the ocular manifestations were concentration-dependent, with 10% CN causing moderate iritis, keratitis, corneal scarring, and neovascularization with minimal resolution (Ballantyne et al., 1975). These results were supported by Gaskins et al., who found that >4% CN dissolved in 1,1,1-trichloroethane caused permanent corneal damage in rabbits (Gaskins et al., 1972).

Oksala et al. described five cases of eye injuries caused by aerosol irritant projections and one by tear-gas pistol (Oksala and Salminen, 1975). In all cases, patients were under the influence of alcohol when the damage occurred. Ocular manifestations were lid and conjunctival erythema, corneal epithelial erosions, stromal edema, Descemet membrane folds, pseudo-pterygium formation, and anterior chamber inflammation. The vision was only partially restored at the last visit since most cases developed corneal opacifications (Oksala and Salminen, 1975). The authors suggest that permanent corneal damage could have resulted from an impaired blinking reflex in drunk patients leading to increased ocular surface exposure and time of contact with the chemical (Oksala and Salminen, 1975). Gerber et al. managed a 2.5-year-old-boy who was accidentally exposed to OC from approximately 30 cm. At presentation, the slit-lamp exam was normal. However, 3 weeks after the incident, the proliferation of conjunctival tissue at the superior and temporal limbus developed and was subsequently removed surgically. Histopathological examination showed mixed acute and chronic inflammation between collagen fibers. The authors hypothesize that the impact on the limbal stem cell niches may have stimulated cellular proliferation and that special vigilance for limbal stem cell deficiency will be required in this case (Gerber et al., 2011).

In animals, milder ocular surface lesions were observed when they were not anesthetized or restrained; thus, their ability to blink was not affected (MacLeod, 1969). Moreover, in all cases, the firing distance was less than 1 m, and immediate management was not given, hindering adequate ocular surface healing (Oksala and Salminen, 1975). Levine and Stahl evaluated 14 human enucleated eyes after tear-gas explosions at close distance (Levine and Stahl, 1968). Five eyes were enucleated 2 months or less after injury due to necrotizing keratitis and suppurative iridocyclitis. The remaining nine eyes, enucleated up to 15 years after insult, exhibited neurotrophic keratopathy (NK), leading to corneal neovascularization, ulceration, and chronic perforation (Levine and Stahl, 1968). Histological analysis revealed epineurium thickening resulting in an impaired sensory activity. The latter is probably due to CN reaction with sulfhydryl protein groups, irreversible enzyme inhibition, and denaturation (Levine and Stahl, 1968). NK results in absent corneal sensation leading to impaired trophic function, corneal epithelial regeneration, and increased risk of infection, ulceration, and perforation (Ruiz-Lozano et al., 2021).

### 5.3 Chlorobenzylidene malononitrile (CS)

#### 5.3.1 Chemical properties

CS is an electrophilic molecule developed in 1928 by the American scientists Corson and Stoughton, hence the abbreviation using the first letters of their last names (Olajos and Salem, 2001). However, it was not used as an RCA until 1958 by the British army in Cyprus, when it replaced CN as a more potent but less toxic alternative for non-lethal crowd control. This crystalline-white powder with a cyanocarbon structure has a melting point of 93ºC and a boiling point of 310ºC. It is slowly hydrolyzed into *o*-chlorobenzaldehyde and malononitrile in water (O’Neil et al., 2006). The half-maximal activation concentration of CS for the TRPA1 channel is EC_50_ CS = 7 ± 1 nM (Bessac et al., 2009).

#### 5.3.2 Mechanism of toxicity

Previously, researchers hypothesized that CS reacted with glutathione, mercapto group-containing enzymes, cysteine thiol groups (present in TRPA1 channels), proteins, and nucleic acids (Olajos and Salem, 2001; Committee on Acute Exposure Guideline Levels et al., 2014). However, it is now known that CS is an agonist of the TRPA1 channel, which facilitates the nerve-ending release of Substance P, CGRP, and other substances after activation (Brône et al., 2008). This elicits neurogenic inflammation and hypersensitivity to mechanical and thermal stimuli as part of the physiological function of these fibers to protect the cornea from noxious cold (Bautista et al., 2006).

#### 5.3.3 Ocular manifestations

The most common manifestations of CS exposure include lacrimation, blepharospasm, irritation, and conjunctivitis, all of which have immediate onset (Kiel, 1997; Davey and Moppett, 2004). Conjunctivitis and tearing can occur even with indirect exposure to the gas, especially if this happens in enclosed spaces (Karaman et al., 2009). Interestingly, some police officers have developed clinical features when handling items contaminated with CS after entering rooms previously occupied by detainees exposed to tear gas. Some cases of contact allergic reactions have been reported where the patients develop dramatic eyelid edema (Watson and Rycroft, 2005). Hill presented a case report of a man directly sprayed with CS on his face, chest, and arms. This patient only developed periorbital edema and conjunctival injection, but he did have more severe respiratory symptoms (Hill et al., 2000). Kiel describes six patients who were affected inside a public house, where all of them only had conjunctival injection and decreased tear break-up time (Kiel, 1997). It seems that CS has less severe clinical manifestations compared to the other tear gases.

### 5.4 Dibenzoxazepine (CR)

#### 5.4.1 Chemical properties

CR is a pale yellow crystalline solid with a melting point of 73ºC. It is not hydrolyzed when in aqueous solutions and has a pepper-like odor. This compound has irritant properties in concentrations of 0.0025% or lower. It has fewer respiratory effects than CS but more pronounced dermatologic consequences. Additionally, it remains longer in the air and on clothing than the other tear gases. Finally, it has a higher lethal median dose than CS. For CR, the half-maximal activation dose is EC50 CR = 308 ± 150 nM (Bessac et al., 2009).

#### 5.4.2 Mechanism of toxicity

Like CS and CN, CR is a potent, selective agonist of the TRPA1 cation channels (Brône et al., 2008). The discovery of the mechanism of actions of the other tear gases was dependent on the study of CR and the structurally similar morphanthridine tricyclic moieties (Gijsen et al., 2010).

#### 5.4.3 Ocular manifestations

The experiment conducted by Ballantyne et al. (1975) in rabbits determined that a solution >5% CR induced transient keratitis in the animals Ballantyne et al., 1975. On the other hand, Rengstorff et al. used 5% CR in propylene glycol 5 days per week for 4 weeks and found only moderate transient conjunctivitis, but no anatomical alterations in the *post mortem* examination of corneal and palpebral structures (Rengstorff et al., 1975).

In humans, CR causes intense blepharospasm, conjunctival irritation and lacrimation when it meets the ocular surface. It is the most potent lacrimator of the RCAs described in this review and has the least systemic toxicity. To this point, Ballantyne and Swanston determined that the concentration required to elicit blepharospasm in humans is lower for CR than it is for CS (Ballantyne and Swanston, 1974). The blepharospasm impedes eye opening, but visual acuity (VA) frequently remains unaffected if patients manage to open their eyes. In cases were sprayed from close range and with highly concentrated preparations, corneal edema, necrotizing keratitis, iridocyclitis, and anterior chamber angle deformities can occur (Leopold and Lieberman, 1971; Blain, 2003).

## 6 Complications

RCAs are associated with ocular surface complications including slow-healing corneal defects, opacification, neovascularization, hypoesthesia, decreased VA, and dry eye disease (DED) (Hoffmann, 1967; Oksala and Salminen, 1975; Epstein and Majmudar, 2001; Holopainen et al., 2003). VA alterations range from transient blurred vision to permanent irregular astigmatism depending on chemical concentration and distance of impact, with some patients recovering almost fully while others do not (Hoffmann, 1967; Kim et al., 2016). In the case of CN, studies in rabbits and monkeys show that directly inoculated animals develop corneal scarring and neovascularization that can persist for months (MacLeod, 1969). In a human study by Rose et al., nine out of 12 cases exposed to CN had epithelial defects that resolved within 3 days, yet three out of 12 patients had confluent corneal punctate staining that remained for 3 weeks, one of which had stromal opacification that persisted for as long as 5 months (Rose, 1969). Uhde describes military cases from World War I. In this report, a patient developed permanent blindness secondary to close-range explosion of a CN grenade detonation, while another who was shot by a tear gas pistol developed corneal edema, hypopyon, and was also left blinded (Uhde, 1948). Some of the more chronic findings in Oksala’s evaluated patients included persistent corneal opacifications, Descemet’s folds, and even a pseudopterygium that reduced VA in one of the patients (Oksala and Salminen, 1975).

Other severe complications can be found in the literature (Midtbo, 1964; Blain, 2003). The report by Levine discusses findings from cases of the Armed Forces Institute of Pathology related to 14 eyes that were enucleated following injury from tear gas weapons (Levine and Stahl, 1968). Half of the cases involved soldiers who accidentally self-inflicted their wounds while examining gas canisters and other devices, while the other half was wounded by a second person (law enforcement officer) who fired with the intent to disable. Five eyes were enucleated within 2 months of the injury, while nine eyes were enucleated between 8 months and 15 years after the inciting event. Medical records indicated that the patients’ corneas were opaque, vascularized, or ulcerated. Notably, anterior chambers of four eyes contained debris, pus, and fibrin was also found, as well as hypopyon. Secondary glaucoma was present in three eyes. Microscopic examination of all eyes revealed intense necrotizing keratitis with deep coagulative necrosis. Iridocyclitis was commonly found along with inflammatory debris, shallowing of the anterior chamber, and retrocorneal membranes (Levine and Stahl, 1968).

It is important to consider that CN is more toxic than CS, as shown in the testing done by Gaskin et al. In this experiment, CN and CS were administered at comparable concentrations (1%–4% and 10%) to unanesthetized rabbit corneas and skin. The rabbits who received CN developed corneal opacities in addition to iritis and conjunctivitis, while those who were received CS developed no enduring corneal injuries (Gaskins et al., 1972). A systematic review identified symptoms like lacrimation, blepharospasm, conjunctivitis, and decreased vision. However, all of these toxic effects were transient and no chronic manifestations were reported (Dimitroglou et al., 2015). Although CS does not produce severe ocular manifestations like the other RCAs commented in this review, it is in fact associated with serious respiratory complications that may necessitate intensive care (Hill et al., 2000).

OC is also associated with the previously mentioned complications as well as conjunctival chemosis, pseudopterygium, and neurotrophic keratitis (Brown et al., 2000; Kniestedt et al., 2005; Voegeli and Baenninger, 2014). One specific case of OC with tardive irrigation led to permanent VA deterioration related to irregular astigmatism and corneal opacification (Epstein and Majmudar, 2001). A report of close-range exposure describes a severe ocular chemical burn that resulted in a pseudopterygium with persistent corneal peripheral conjunctivalization 6 months post-exposure. This patient presented with a corneal erosion and microhyphema which were treated topical corticosteroids, antibiotics, and autologous serum tears. However, the erosion persisted in subsequent consultations, and after 4 weeks, slit lamp examination revealed the pseudopterygium with corneal neovascularization suggestive of limbal necrosis. Conjunctivalization was still present at 6-months post initial evaluation (Voegeli and Baenninger, 2014). DED is also a significant long-term complication, as demonstrated in a study evaluating the decrease in aqueous tear production following pepper spray exposure. In this report, 96 patients who were exposed to OC during the Gezi Parks protests in Turkey evaluated for DED using Schirmer’s test and the Dry Eye Questionnaire (DEQ). All patients were treated by irrigation with alkaline substances (milk and antacid solutions). Additionally, 82 individuals reported using protective goggles during the episode. The authors determined statistically significant differences between Schirmer’s I and II between those who used goggles and those who did not (3.21 ± 1.55 to 8.24 ± 1.24 mm *p* < 0.001; and 5.15 + 1.5 to 13.2 ± 1.66 mm *p* < 0.001, respectively). Additionally, 24.4% and 35.7% of those who did and did not wear goggles reported symptoms in the DEQ (Rasier et al., 2015).

Other studies mention non-ocular surface manifestations like with cataracts, glaucoma, vitreous hemorrhage, and optic nerve damage (Hoffmann, 1967). However, traumatic injury is most likely the culprit of these complications, because patients from these reports were exposed in the context of explosive devices that cause blasts, shock-wave damage or direct impact from tear gas cannisters. A summary of the mechanism of action, ocular manifestations, and complications caused by RCA exposure is presented in Table 1 of this review.

**TABLE 1:** Mechanism of action and ocular manifestations of Riot Control Agents.

RCA	Toxic mechanisms	Acute ocular manifestations	Complications	References
*Oleoresin capsicum* (OC)	TRPV1 agonism	Blepharospasm, ocular pain, conjunctival injection, PEE	Symblepharon, chemosis, pseudopterygium, persistent corneal conjunctivalization	Brown et al. (2000), Zollman et al. (2000), Epstein and Majmudar, (2001), Holopainen et al. (2003), Das et al. (2005), Kniestedt et al. (2005), Voegeli and Baenninger, (2014), Rasier et al. (2015)
			DED, Neurotrophic Keratitis, irregular astigmatism, and corneal opacification	
		Corneal hypoesthesia and decreased tear production		
*Chloroacetophenone* (CN)	TRPA1 agonism	Periocular erythema, PEE, corneal stromal edema, Descemet’s folds, and anterior chamber inflammation	Pseudopterygium formation, conjunctival proliferation	Uhde, (1948), Levine and Stahl, (1968), Rose, (1969), Oksala and Salminen, (1975), Gerber et al. (2011), Dimitroglou et al. (2015)
			Iridocyclitis	
			Secondary glaucoma	
			Blindness	
		Neurotrophic keratitis with corneal neovascularization, ulceration, and perforation		
*Chlorobenzylidene malononitrile* (CS)	TRPA1 agonism	Lacrimation, blepharospasm, conjunctivitis, allergic reactions, eyelid edema	Not associated with chronic ocular manifestations or complications	Gaskins et al. (1972), Kiel, (1997), Hill et al. (2000), Davey and Moppett, (2004), Watson and Rycroft, (2005)
*Dibenzoxazepine* (CR)	TRPA1 agonism	Most potent lacrimator of the RCAs, blepharospasm with lower concentrations	Corneal edema, necrotizing keratitis, iridocyclitis	Levine and Stahl, (1968), Leopold and Lieberman, (1971), Ballantyne and Swanston, (1974), Blain, (2003)
		Corneal edema, necrotizing keratitis, iridocyclitis		
			Anterior chamber angle deformation	

DED, Dry eye disease; PEE, Punctate epithelial erosions; RCA, Riot control agent; TRPA1, Transient receptor potential ankyrin 1; TRPV1, Transient receptor potential vanilloid 1.

## 7 Management of exposure to RCAs

### 7.1 Decontamination

The management of patients exposed to tear gas should begin immediately with field decontamination. First and foremost, it is vital that physicians avoid their own contamination and that of their equipment. This can be done by wearing protective eyewear, surgical masks, and gowns. Patients should be lifted off the ground and be treated in well ventilated spaces, as tear gas particles can accumulate easily (Carron and Yersin, 2009; Schep et al., 2015). Contact lenses should be removed if appropriate. The most important step is irrigation with water or normal saline for 15–20 min to remove tear gas particles from the ocular surface (Breakell and Bodiwala, 1998; Blain, 2003; Carron and Yersin, 2009). In those with pronounced blepharospasm, topical anesthetics can facilitate eye-opening for irrigation of the superior and inferior *cul-de-sacs*, where the chemicals may accumulate. Historically, some authors recommended blowing air into the patient’s eyes, but it has been determined that this technique may worsen the symptomatology by dispersing tear gas to unaffected areas (Breakell and Bodiwala, 1998; Gray, 2000).

### 7.2 Ophthalmological evaluation

Patients with moderate-severe ocular symptoms warrant referral to an ophthalmologist for comprehensive evaluation (Kearney et al., 2014). The initial assessment by the ophthalmologist must include an account of the exposure, to determine its duration, whether it was direct or indirect, and distance from which the substance was fired. VA should be obtained using a Snellen chart at 20 feet inside a dim room to establish a baseline measurement. Patients with reduced VA after exposure warrant a thorough ophthalmic evaluation.

The slit lamp exam to search for conjunctival hyperemia, chemosis, and skin inflammation. Lissamine green staining should preferentially be used to determine the extent of conjunctival epithelium damage. A corneal exam should include an evaluation of its epithelial integrity, with fluorescein staining helping to detect epithelial erosions (Brown et al., 2000). Fluorescein can also aid to find corneal ulcers, especially in patients presenting with severe pain and hyperemia (Shimada et al., 2012). The anterior chamber should be carefully evaluated for signs of an inflammatory response, such as cells and flare. Visibly embedded RCA particles can be removed under the biomicroscope with a cotton swab or a needle (Blain, 2003).

It is important to evaluate corneal sensation in follow-up visits, after the acute symptoms have subsided. Patients with hypoesthesia are predisposed to develop corneal ulcers, especially if they engage in eye rubbing (Holopainen et al., 2003; Shimada et al., 2012). The Cochet Bonnet Esthesiometer is an instrument used to measure the corneal sensitivity threshold and can help clinicians detect nerve damage. It consists of a nylon monofilament with variable length that is used to touch the central and peripheral corneal, where the patient’s blinking is considered a positive response. The test should begin using the full length of the filament (60 mm) and continue with 5-mm decreases until a positive response is obtained. Additionally, another objective measurement to evaluate corneal nerve integrity is *in vivo* confocal microscopy, which allows for the direct visualization of the fibers (Holopainen et al., 2003). The combination of both techniques provides a thorough examination of corneal sensory function.

### 7.3 TRP inhibitors

The TRPA1 channel is the primary driver of the tissue response after CS, CN, and CR exposure and its concomitant release of inflammation-inducing neuropeptides. Therefore, some studies investigating potential drugs to block the TRPA1 channels show promise as a treatment for the hazardous health effects of RCAs. Although none of these compounds are approved for the treatment of RCA exposure in humans, previous *in vitro* and animal studies have had success blocking their effects.

For example, the study by Bessac et al. proved that TRPA1-mediated Ca^2+^ influx mediates the toxic effects of CS, CN, and CR; and that genetic ablation or pharmacological inhibition of the TRPA1 channel deterred CS or CN-induced nocifensive behavior. The *in vitro* results from this experiment determined the half-maximal activation concentrations for CS, CN, and CR, which were mentioned previously in each agent’s subsection. For the *in vivo* section, one group of genetically ablated TRPA1−/−mice and another group of wild-type animals receiving the first-generation TRPA1 antagonist *HC-030031* were exposed to CS and CN (100 mM dosage) *via* ocular or dermal routes. The genetically ablated mice failed to perceive the tear gases as noxious agents, demonstrated by a total abolishment of response after their administration. On the other hand, the wild-type, pharmacologically treated mice had reduced nocifensive responses after applying the TRPA1 antagonist (Bessac et al., 2009). Based on these findings, the authors suggested that *HC-030031* reduces the acute sensory irritation induced tear-gas mediated TRPA1 pathway activation.

In humans, biopharmaceutical companies have performed early-phase clinical trials investigating the effectiveness of TRPA1 antagonism in other clinical conditions, such as neuropathic pain and allergic asthma. A phase two randomized, controlled, double-blinded clinical trial evaluating the TRPA1 blocker ISC-17536 (Glenmark Pharmaceuticals) as monotherapy for painful diabetic neuropathy was published recently (Agarwal et al., 2014). *ISC-17536* did not show significant efficacy in treating diabetic peripheral neuropathy. Still, the authors hypothesize that since the pharmacological site of action is on small peripheral nerve fibers, patients who have lost these neurons are unlikely to respond to TRPA1 inhibition. However, an effect could be seen in those who have preserved small nerve fibers (Jain et al., 2022). Clinical trials studying other molecules, such as *HX-100*, *GDC-0334,* and *ODM-108,* for allergic asthma and neuropathic pain have halted due to unfavorable pharmacokinetics (Chen and Terrett, 2020; Souza Monteiro De Araujo et al., 2020).

TRPV 1 blockade may also effectively eliminate ocular symptoms of pain. Although TRPV 1 blockade in RCA exposure has not been studied, it has reduced ocular pain and inflammation in other clinical contexts. For example, in a murine model developed by Fakih et al., DED mice who received topical TRPV1 antagonist capsazepine twice daily for 2 weeks showed inhibition of commonly upregulated genes involved in inflammatory and neuropathic pain. This experiment also demonstrated a reduced sensation of ocular pain (Fakih et al., 2021). In another model for allergic keratoconjunctivitis, pretreatment of mice with TRPV1 antagonists reduced the inflammatory reaction and prevented sensitization of nociceptors, resulting in decreased ocular pain (Callejo et al., 2015).

### 7.4 Chelating agents

Diphoterine^®^ solution (Prevor Laboratory, Valmondois, France) is a hypertonic, amphoteric, and chelating substance recommended for dermal or ocular exposure to various chemicals (Gerard et al., 2002; Rihawi et al., 2006; Dohlman et al., 2011). This compound has six binding sites that allow clearance of different substances, including acids, bases, and alkylating agents, among others (Hall et al., 2002). Gerard et al. successfully managed a severe ocular chemical burn patient after rinsing the eye with 1 L of Diphoterine and prevented the development of sequelae (Gerard et al., 2002). Importantly, Diphoterine rinsing reduced the patient’s stromal edema, a risk factor that has been correlated with the severity of subsequent leucomas (Kubota and Fagerholm, 1991). Viala et al. conducted an experiment in which five French Gendarmes voluntarily entered a chamber with CS concentrations of 3,000 mg/m^3^. Four of them quickly developed incapacitating symptoms, which resolved in 4 min after exiting the chamber and being decontaminated with 250 mL of Diphoterine. One of the Gendarmes applied the solution before entering the chamber, and his only symptom was mild cough that also resolved after a few minutes (Viala et al., 2005). The results that Brvar obtained years later further support the use of Diphoterine, in an experiment where Slovenian police officers in training ran for 20 s through a cloud generated from CS grenades in an open field. Officers who sprayed Diphoterine on themselves before CS exposure had lower levels of facial pain and were more rapidly able to return to duty. Those treated with the solution after the exercise also recovered from their symptoms, albeit not as quickly; finally, those who did not receive treatment reported the highest levels of pain and were incapacitated the longest (Brvar, 2016).

## 8 Conclusion

RCAs can be more harmful than initially thought. Although, in many cases, the classic manifestations of conjunctivitis, lacrimation, and blepharospasm are transient, this review reveals that there can be much more severe sequelae that necessitate ophthalmological referral and follow-up. Much of the available literature comes from case reports and case series. These documents have a low level of epidemiological significance. Still, the complications described in them should alert the medical community about the need for more in-depth knowledge of the effects of tear gases. Additionally, the basis of care currently rests upon decontamination through irrigation. Some efforts to develop specific antidotes have been taken, but none have yet been successfully released into the public. To completely understand the long-term sequelae, more thorough research efforts are needed and to develop target-specific treatments. Formal therapeutic guidelines should be implemented to standardize the treatment of exposed patients and protect the medical team from contamination.
